# RhoA/ROCK signaling as a potential convergent pathway in microbiota–gut–brain axis-related cognitive impairment

**DOI:** 10.3389/fneur.2026.1909220

**Published:** 2026-08-24

**Authors:** Yang Cao, Xingquan Wu, Junjie Yao, Huijuan Lou, Yahui Sun, Jinglei Jiang, Ya Song, Xia Meng, Chenyang Du, Daichaozi Da, Deyu Cong, Yuanyuan Ji

**Affiliations:** 1 School of Acupuncture and Tuina, Changchun University of Chinese Medicine, Changchun, China; 2 Department of Tuina, Affiliated Hospital of Changchun University of Chinese Medicine, Changchun, China; 3 Department of Tuina, Guang'anmen Hospital of China Academy of Chinese Medical Sciences, Beijing, China

**Keywords:** cognitive impairment, gut microbiota, neuroinflammation, RhoA/ROCK signaling pathway, synaptic plasticity

## Abstract

Cognitive impairment is increasingly recognized as a multifactorial neurological condition involving gut microbiota imbalance, impaired barrier function, neuroinflammatory activation, and disrupted synaptic plasticity. However, the molecular mechanisms that integrate these pathological events remain largely unclear. The RhoA/ROCK signaling pathway, a key regulator of actin cytoskeleton remodeling, intercellular junction dynamics, barrier permeability, inflammatory signaling, and neuronal structural plasticity, may represent a potential molecular interface connecting intestinal microbial disturbances with cognitive dysfunction. Accumulating evidence suggests that alterations in gut microbiota composition, microbiota-derived metabolites, and inflammation-related microbial signals can modulate RhoA/ROCK activity, thereby affecting intestinal barrier integrity and blood–brain barrier function, regulating systemic and neuroinflammatory responses, and influencing cognition-related processes, including synaptic remodeling, axonal guidance, and long-term potentiation. Nevertheless, the biological consequences of RhoA/ROCK activation are highly dependent on cellular and pathological contexts. Transient and spatially restricted activation may contribute to barrier restoration and synaptic maintenance, whereas persistent or excessive activation may promote barrier disruption and neuronal dysfunction. Although current evidence remains largely preclinical and heterogeneous, RhoA/ROCK signaling provides a potential mechanistic framework for understanding microbiota–gut–brain interactions in cognitive disorders. Further investigations are required to establish causal relationships and clarify its therapeutic relevance.

## 1 Introduction

The incidence of cognitive impairment increases markedly with advancing age. In the context of rapid global population aging and the limited availability of effective therapeutic strategies, cognitive impairment has emerged as a major public health challenge worldwide (1). Beyond imposing substantial physical and psychological burdens on affected individuals, cognitive impairment also generates considerable socioeconomic pressure on families and healthcare systems. From a pathological perspective, cognitive decline is closely associated with neuroinflammatory responses, oxidative stress, and other pathological cascades triggered by abnormal protein accumulation in the brain, ultimately resulting in neuronal degeneration and disruption of protective brain barrier systems. Recent studies have further highlighted the critical regulatory role of the microbiota–gut–brain axis in cognitive function (2).

The microbiota–gut–brain axis represents a complex bidirectional communication network linking intestinal microorganisms and the central nervous system through neural, immune, endocrine, and metabolic pathways (3). Gut microbiota influence host physiological and pathological processes through regulation of vagal signaling (4), the enteric nervous system (5), microglial activity, and the hypothalamic–pituitary–adrenal axis (6). In addition, microbiota-derived metabolites, including short-chain fatty acids (SCFAs) and tryptophan-derived compounds, participate in host regulation (7). Disruption of these interactions may impair intestinal barrier function, increase intestinal permeability, and subsequently compromise blood–brain barrier integrity, thereby affecting central nervous system homeostasis. Furthermore, impaired microbiota–gut–brain communication may facilitate abnormal aggregation of *β*-amyloid (8) and *α*-synuclein (9), contributing to the initiation and progression of cognitive impairment associated with Alzheimer’s disease and Parkinson’s disease.

Despite growing recognition of the microbiota–gut–brain axis in cognitive disorders, the precise molecular mechanisms through which gut microbiota dysbiosis induces barrier dysfunction, inflammatory signaling, and alterations in central nervous system function remain incompletely understood. RhoA, a member of the Ras homologous (RHO) GTPase family, is a crucial regulator of cytoskeletal organization and participates in diverse cellular processes, including migration, proliferation, and survival (10). Given that the RhoA/ROCK signaling pathway regulates barrier stability, inflammatory responses, and neuronal plasticity, it has attracted increasing attention as a potential molecular link between microbial disturbances and cognitive dysfunction.

Previous studies have provided preliminary evidence supporting this hypothesis. Ding et al. (11) demonstrated that RhoA/ROCK signaling contributes to the reciprocal aggravation between cerebral ischemia–reperfusion injury and colitis. Using genome-wide association analysis, Las et al. (12) identified RHOA as a susceptibility-associated gene involved in inflammatory bowel disease and suggested a potential relationship between RHOA-related signaling and hypothalamic-like neuronal populations (13).

Although accumulating evidence indicates that intestinal dysbiosis, barrier impairment, and neuroinflammation collectively contribute to cognitive decline, the molecular mechanisms integrating these pathological processes remain insufficiently characterized. RhoA/ROCK signaling may function as a regulatory convergence point that connects microbiota-derived signals, barrier homeostasis, and neuroimmune responses. Mechanistically, microbial metabolites or inflammation-associated molecules generated during dysbiosis may regulate RhoA/ROCK activity, thereby altering intestinal epithelial barrier function and blood–brain barrier integrity. Subsequent barrier disruption may facilitate the entry of peripheral inflammatory signals into the central nervous system, influencing microglial activation, neuroinflammatory responses, and synaptic plasticity.

However, direct evidence demonstrating a complete causal pathway of “microbiota alteration–RhoA/ROCK dysregulation–cognitive impairment” remains limited. Therefore, this review does not regard RhoA/ROCK as an established central mediator of microbiota–gut–brain axis-associated cognitive dysfunction. Instead, it evaluates RhoA/ROCK as a biologically plausible convergent signaling pathway and examines its potential involvement in multiple pathological processes. Importantly, RhoA/ROCK activity is highly dependent on cell type, temporal and spatial regulation, and isoform-specific functions. Alzheimer’s disease, vascular cognitive impairment, and Parkinson’s disease-related cognitive decline involve distinct upstream pathological mechanisms; however, comparative analyses addressing both common and disease-specific roles of RhoA/ROCK remain limited. Moreover, the therapeutic feasibility, safety, and isoform selectivity of broad RhoA/ROCK inhibition require further investigation.

Accordingly, this review aims to integrate current evidence regarding RhoA/ROCK-mediated regulation of barrier integrity, inflammatory responses, and neuronal plasticity within the microbiota–gut–brain axis. Specifically, we distinguish established mechanisms from hypothetical associations, compare the pathological significance of RhoA/ROCK signaling across different cognitive disorders, and evaluate the reproducibility, species relevance, and translational limitations of existing findings.

## 2 The microbiota–gut–brain Axis and cognitive impairment

For many years, research on cognitive impairment has primarily focused on pathological alterations occurring within the central nervous system. The emergence of the microbiota–gut–brain axis concept, however, has broadened this perspective by emphasizing the role of intestinal microbial regulation in shaping brain function and cognitive processes. This axis represents a dynamic bidirectional communication system in which neural, immune, endocrine, and metabolic pathways collectively mediate interactions between the gastrointestinal tract and the brain (3).

Early investigations into the relationship between gut microbiota and cognition demonstrated that microbial disruption does not inevitably lead to uniform cognitive outcomes. Instead, the consequences of microbiota depletion or alteration appear to vary according to experimental design, host characteristics, and biological context. For example, antibiotic-induced gut microbiota dysbiosis in adult mice was associated with impaired recognition memory (14). In contrast, germ-free C57BL/6 J mice exhibited age- and paradigm-dependent changes in spatial and contextual memory performance (15). Moreover, transplantation of fecal microbiota from aged donor mice into young recipients resulted in cognitive impairment accompanied by hippocampal synaptic loss (16). Together, these findings support the notion that gut microbiota contributes to cognitive regulation; however, they do not indicate that microbiota depletion itself is sufficient to induce persistent cognitive decline.

Cognitive impairment is frequently observed in neurodegenerative and cerebrovascular disorders, particularly Alzheimer’s disease (AD), vascular dementia, and Parkinson’s disease (PD). Despite their distinct pathological origins, these disorders share overlapping manifestations of cognitive dysfunction. In AD, progressive memory decline represents the most prominent clinical feature (17). Cognitive deficits associated with vascular dementia are primarily attributed to cerebrovascular damage and subsequent impairment of neuronal function (18). In PD, dementia generally develops during later disease stages and is characterized by impairments in attention, executive function, and visuospatial abilities (19).

A growing body of evidence suggests that RhoA/ROCK signaling may contribute to cognitive impairment across these disorders. By regulating barrier integrity, inflammatory activity, and neuronal plasticity, this pathway may influence multiple pathological processes associated with cognitive decline. Nevertheless, its involvement in microbiota–gut–brain axis-mediated cognitive dysfunction remains largely based on indirect observations, and direct causal relationships have not yet been conclusively demonstrated.

In AD, Wang et al. (20) transplanted fecal microbiota derived from six patients with AD or APP/PS1 transgenic mice into wild-type recipient mice. This approach resulted in alterations in microbial composition and increased cortical markers associated with endoplasmic reticulum stress, whereas inhibition of trimethylamine N-oxide (TMAO) production partially reversed these molecular abnormalities. However, behavioral assessments of cognitive function were not conducted in this study. Therefore, these findings provide evidence for the transmission of microbiota-associated molecular phenotypes but do not establish that cognitive impairment itself can be transferred through microbiota transplantation. In parallel, multiple studies have reported gut microbiota alterations in patients with AD and AD animal models, although whether these microbial changes actively contribute to disease initiation or progression remains unresolved (21, 22).

The functional effects of microbiota-derived metabolites also appear to be highly dependent on physiological and pathological contexts. Short-chain fatty acids (SCFAs) are generally considered beneficial microbial metabolites due to their barrier-protective and anti-inflammatory properties. However, supplementation with an SCFA mixture in germ-free AD model mice unexpectedly increased Aβ plaque deposition and altered microglial phenotypes. These observations indicate that the biological consequences of SCFAs are not universally protective but may instead depend on factors such as disease stage, host background, and local tissue environment (23).

In PD, increasing evidence suggests that gut microbiota alterations may participate in disease progression and the regulation of non-motor symptoms. Individuals with PD frequently exhibit microbial dysbiosis characterized by reduced abundance of SCFA-producing bacteria and enrichment of inflammation-associated microbial populations (19). Mechanistic studies have proposed several pathways through which microbiota alterations may influence PD pathology, including modulation of intestinal barrier function, peripheral immune activation, and microbial metabolite production. For instance, microbiota-derived inflammatory signals may facilitate abnormal *α*-synuclein aggregation, whereas microbial metabolites may regulate microglial activation and neuroinflammatory responses. However, whether gut microbiota changes directly initiate PD pathology and contribute specifically to PD-associated cognitive impairment remains uncertain due to the lack of sufficient causal evidence (9).

Vascular cognitive impairment (VCI) is closely associated with cerebral small vessel disease, blood–brain barrier (BBB) dysfunction, and persistent neuroinflammatory responses. Gut microbiota may influence VCI progression by altering systemic metabolic balance and immune homeostasis. Changes in microbial composition have been observed in patients with VCI as well as in experimental models of cerebrovascular disorders. Microbiota-derived metabolites may further affect cognitive outcomes through regulation of inflammatory pathways, oxidative stress, and vascular function (18).

Among microbial metabolites, SCFAs have been reported to strengthen intestinal and BBB integrity and exert potential anti-inflammatory and neuroprotective effects. Conversely, trimethylamine N-oxide (TMAO) may promote cerebrovascular injury by inducing oxidative stress, endothelial dysfunction, and neuroinflammation (18, 24). Nevertheless, current evidence is mainly derived from animal studies and observational clinical analyses, and the molecular mechanisms underlying the influence of microbial metabolites on VCI remain incompletely understood. Interventions targeting gut microbiota dysbiosis, including probiotics, prebiotics, and synbiotics, may reshape microbial communities and modulate both gastrointestinal and central nervous system functions, suggesting potential therapeutic relevance for cognitive impairment associated with AD and PD (25, 26).

Overall, the microbiota–gut–brain axis regulates cognitive function through an interconnected network involving neural, immune, endocrine, and metabolic pathways. Although current research has highlighted the importance of mechanisms involving the vagus nerve, SCFAs, and tryptophan-derived metabolites, the precise molecular interactions and regulatory networks underlying these processes remain to be fully clarified.

Role of the RhoA/ROCK Signaling Pathway in the Microbiota–Gut–Brain Axis.

### 2.1 RhoA/ROCK signaling pathway

The Rho GTPase family constitutes a subgroup of the Ras superfamily of small GTPases, among which RhoA is one of the most extensively investigated members (27). Rho-associated coiled-coil-containing protein kinases (ROCKs) are serine/threonine kinases belonging to the AGC (PKA/PKG/PKC) kinase family and function as the primary downstream effectors of RhoA signaling. The ROCK family contains two isoforms, ROCK1 and ROCK2, which exhibit approximately 65% overall sequence homology and 92% identity within their catalytic kinase domains (28).

RhoA/ROCK signaling serves as a central regulator of actin cytoskeletal dynamics and participates in the control of cellular morphology, migration, and intercellular junction organization through actomyosin contractility. The activity of RhoA is tightly regulated through reversible cycling between an inactive GDP-bound state and an active GTP-bound state. Under physiological resting conditions, RhoA is predominantly maintained in its GDP-bound form. Following extracellular stimulation, guanine nucleotide exchange factors (GEFs) facilitate GDP release and promote GTP loading, thereby converting RhoA into its active state and initiating downstream ROCK activation. In contrast, GTPase-activating proteins (GAPs) accelerate GTP hydrolysis, returning RhoA to its inactive GDP-bound form and terminating signal transduction (29). Rho GDP dissociation inhibitors (GDIs) further fine-tune RhoA activity by stabilizing the RhoA–GDP complex and regulating its intracellular distribution, thereby ensuring the spatial and temporal control of RhoA signaling (30).

Importantly, the biological consequences of RhoA activation are determined not only by the overall activation level but also by its subcellular localization and the formation of specific signaling complexes. For instance, transient RhoA activation at intercellular junctions facilitates ZO-1 recruitment and contributes to barrier restoration, whereas persistent or excessive activation promotes cytoskeletal hypercontractility, enhances inflammatory signaling, and compromises barrier integrity (31). Thus, the coordinated actions of GEFs, GAPs, and GDIs, together with the dynamic regulation of RhoA activation patterns, ultimately determine whether RhoA/ROCK signaling produces protective or pathological effects under different cellular and disease conditions.

### 2.2 Interactions between the RhoA/ROCK signaling pathway and the microbiota–gut–brain axis

Microbiota-derived metabolites, pathogen-associated molecular patterns (PAMPs), and microbial toxins have been shown to influence RhoA/ROCK signaling in both intestinal and central nervous system environments, thereby affecting epithelial barrier integrity, neuroinflammatory responses, and cognitive-related processes. However, these regulatory effects are highly dependent on specific microbial species, metabolites, receptors, and cellular contexts. Furthermore, most available evidence originates from *in vitro* systems and animal models, and the complete causal pathway connecting microbial alterations with RhoA/ROCK activation along the gut–brain axis remains to be fully established.

Within the intestine, *Escherichia coli* Nissle 1917 has been reported to maintain epithelial barrier function in Caco-2 monolayers and reduce systemic inflammation in mouse models by inhibiting TLR4-dependent activation of the RhoA/ROCK2/myosin light chain (MLC) signaling cascade (32, 33). Microbial-derived beneficial metabolites, particularly short-chain fatty acids (SCFAs), may contribute to cytoskeletal stability and tight junction preservation by preventing excessive RhoA activation (7). In addition, GroEL and transaldolase (TAL) proteins derived from Bifidobacteria have been shown to modulate RhoA/ROCK signaling, suppress inflammatory cytokine production, enhance tight junction protein expression, and protect intestinal epithelial cells from lipopolysaccharide (LPS)-induced barrier disruption (34). A probiotic combination consisting of Limosilactobacillus fermentum HF07 and *Lactococcus lactis* HF08 has also demonstrated protective effects against inflammation and intestinal barrier impairment in D-galactose/DSS-induced aging colitis mice, potentially through suppression of key RhoA/ROCK pathway components (35). Collectively, these findings suggest that microbial-derived factors may regulate intestinal barrier homeostasis through RhoA-dependent mechanisms involving immune modulation and maintenance of junctional stability.

In the central nervous system, microbiota-derived signals may influence RhoA/ROCK-associated pathways indirectly through inflammatory mediators, microbial metabolites, and changes in barrier function. Microbial components, including PAMPs, exotoxins, and metabolites such as SCFAs, can affect CNS physiology through multiple signaling routes. Microbial metabolite taurodeoxycholic acid has been shown to promote tissue residency and barrier protection of intestinal innate lymphoid cells through the P2Y10–Ca^2+^–RhoA signaling pathway. However, these findings cannot be directly extrapolated to deoxycholic acid or other bile acids, and cognitive outcomes were not evaluated in this study (36). PAMPs commonly activate Toll-like receptors (TLRs), resulting in NF-κB activation and subsequent production of inflammatory mediators, which may stimulate Rho guanine nucleotide exchange factors (RhoGEFs) (37). In contrast, Clostridioides difficile toxins A (TcdA) and B (TcdB) can directly enter host cells, inhibit RhoA activity, and interfere with cytoskeletal organization and tight junction stability (38).

The influence of individual SCFAs on RhoA/ROCK signaling may differ considerably. Animal studies have indicated that valerate suppresses RhoA/ROCK1 signaling through activation of hippocampal GPR41, thereby reducing neuroinflammation and alterations associated with neuronal ferroptosis. However, butyrate does not appear to produce comparable effects on the RhoA/ROCK1 pathway, and direct evidence demonstrating that RhoA/ROCK activation is responsible for cognitive improvement remains insufficient (39). Moreover, cell-free supernatants obtained from *Pediococcus pentosaceus* LAB6 and Lactiplantibacillus plantarum LAB12 were reported to decrease intracellular RhoA activity and reduce F-actin stress fiber formation in SK-N-SH human neuroblastoma cells, as measured by the G-LISA RhoA activation assay (40). LPS-induced activation of RhoA/ROCK signaling in brain endothelial cells primarily occurs through TLR4-dependent mechanisms involving PKC or GEF-H1, leading to disruption of tight junction structures and increased BBB permeability. Nevertheless, evidence from cognitive intervention studies or mechanistic rescue experiments remains limited (41).

Tryptophan-derived indole metabolites mainly regulate intestinal epithelial function, neural progenitor activity, and neuroimmune responses through activation of the aryl hydrocarbon receptor (AhR) pathway (42, 43). Although animal studies have provided substantial evidence supporting their involvement in neurogenesis and cognition-related phenotypes, a direct mechanistic connection between these metabolites and RhoA/ROCK signaling has not yet been demonstrated.

**Figure 1:**
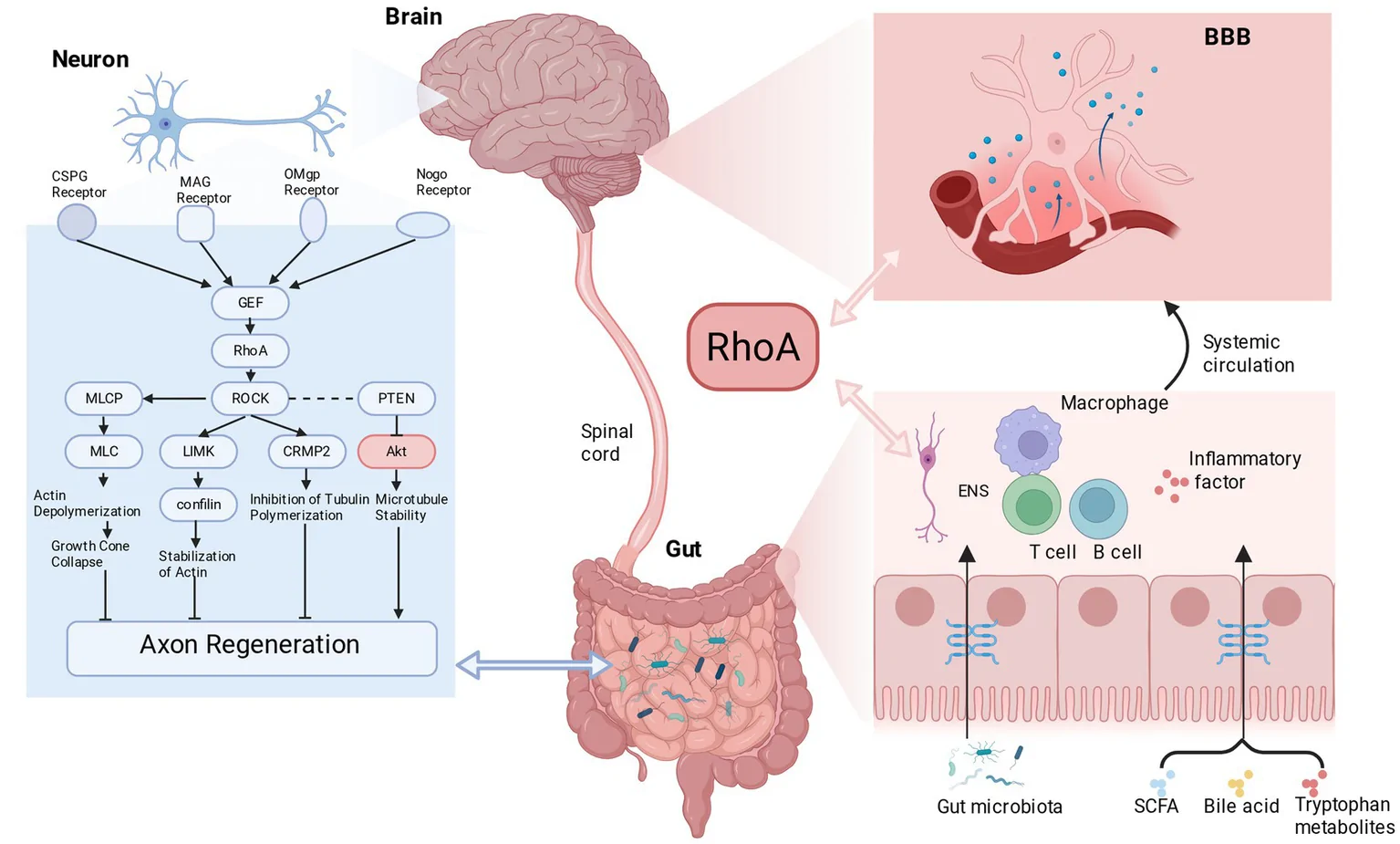
Regulation of the microbiota–gut–brain axis by RhoA/ROCK signaling.

Overall, current studies indicate that microbiota-derived signals can regulate RhoA/ROCK activity across diverse cell populations within the intestine and brain. However, the functional consequences of this regulation vary according to the specific microbial factors involved, receptor pathways, and cellular environments. Fecal microbiota transplantation from aged donor mice into young recipients impaired spatial learning and memory while altering hippocampal proteins associated with synaptic plasticity and neurotransmission, providing relatively direct evidence that microbial alterations can influence central synaptic processes (44). Other investigations suggest that microbiota changes may affect neural regulation through Sema3A-associated axon guidance pathways accompanied by alterations in RhoA/ROCK-related molecules (45). As summarized in Figure 1, microbiota-derived signals may regulate intestinal barrier function, BBB integrity, and neuroinflammatory responses through modulation of RhoA/ROCK activity.

With the emergence of multi-omics technologies and cell-specific analytical approaches, increasing attention has been directed toward understanding how microbial-derived metabolites influence host RhoA/ROCK signaling. However, current studies remain concentrated primarily on intestinal epithelial cells, brain vascular endothelial cells, and immune cell populations. Direct evidence demonstrating that microbial metabolites can alter RhoA/ROCK activity within the CNS and subsequently regulate cognitive behavior remains scarce (46, 47).

Several limitations should be considered when interpreting current evidence regarding the regulation of CNS function by gut microbial signals through RhoA/ROCK signaling, particularly concerning reproducibility, species differences, and causal mechanisms. First, most studies rely on Caco-2, IEC-6, and other immortalized or tumor-derived intestinal epithelial cell models, neuroblastoma systems, or animal models involving colitis, stress exposure, or germ-free conditions. Independent confirmation across diverse experimental platforms remains limited. These models cannot fully capture the complexity of human intestinal barriers, mature neuronal networks, or integrated physiological interactions, and therefore extrapolation to human disease requires careful consideration.

Second, microbial modulation of RhoA/ROCK signaling displays considerable specificity with respect to bacterial strains, metabolites, receptor pathways, and responding cell types. For example, the beneficial effects observed with *E. coli* Nissle 1917 should not be generalized to all *E. coli* strains or probiotic organisms. Moreover, interventions such as probiotic combinations, microbial supernatants, and fecal microbiota transplantation simultaneously influence immune regulation, metabolism, neuroendocrine signaling, and vagal pathways. Therefore, alterations in RhoA/ROCK activity alone cannot establish that this pathway is an essential mediator of the observed biological outcomes.

Importantly, current research mainly demonstrates three separate observations: microbial alterations influence barrier properties, microbial metabolites regulate inflammatory responses, and RhoA/ROCK participates in barrier and inflammatory regulation. However, direct evidence showing that microbiota-derived signals can pass through the “gut–circulation–brain” route, reach the CNS, and directly activate RhoA/ROCK signaling in brain endothelial cells, glial cells, or neurons within cognitive impairment models is still lacking. Most available studies have identified regulatory effects in peripheral barrier-related cells, including intestinal epithelial cells and brain microvascular endothelial cells, whereas the mechanisms responsible for gut–brain signal transmission and their relationship with cognitive outcomes remain unresolved.

Future research should determine whether microbiota alterations directly influence cognition through RhoA/ROCK-dependent mechanisms and further evaluate the translational significance of this pathway using human-derived intestinal–BBB co-culture systems, organoid models, and clinical samples.

### 2.3 Regulation of intestinal barrier and blood–brain barrier by RhoA/ROCK signaling

Although the intestinal barrier and the blood–brain barrier (BBB) serve distinct physiological purposes, both rely on highly organized intercellular junctional structures to maintain selective permeability (48). The intestinal barrier consists of epithelial cells, the mucus layer, immune components, and junctional complexes, functioning as a critical interface that preserves host–microbiota homeostasis by restricting excessive penetration of bacterial products, toxins, and abnormal immune signals into the circulation. When gut microbiota imbalance compromises this barrier, circulating levels of lipopolysaccharide (LPS), inflammatory cytokines, and microbial-derived metabolites may increase, thereby contributing to systemic inflammatory responses (49–51). In comparison, the BBB is primarily composed of brain microvascular endothelial cells, pericytes, astrocytic endfeet, and the basement membrane, and maintains central nervous system (CNS) homeostasis through precise regulation of molecular exchange between the blood and brain parenchyma. Impaired BBB integrity facilitates the infiltration of inflammatory mediators, immune cells, and neurotoxic molecules into the brain, consequently promoting glial activation and amplifying neuroinflammatory processes (52–54).

Within the microbiota–gut–brain axis, these two barriers should be viewed as interconnected components rather than independent structures. They participate in a coordinated regulatory network mediated by microbial metabolites, immune signaling, and neuroendocrine pathways. RhoA/ROCK signaling may represent an important molecular regulator within this network by controlling epithelial cytoskeletal remodeling and intestinal barrier stability, while simultaneously influencing endothelial contractility, tight junction organization, and inflammatory cell migration in the BBB. From this perspective, RhoA/ROCK signaling may serve as a potential interface linking microbiota-associated alterations to central neuroinflammatory responses. Nevertheless, the mechanisms through which microbial factors regulate intestinal and BBB functions remain incompletely understood, and the specific contribution of RhoA/ROCK signaling requires further clarification. Microbial-derived metabolites, including short-chain fatty acids (SCFAs) and bile acids, have been implicated in microbiota–gut–brain communication through their effects on barrier integrity, inflammatory regulation, and vascular homeostasis (55). However, although SCFAs have been shown to influence both intestinal barrier and BBB function, whether they directly regulate RhoA/ROCK activity remains uncertain (51, 52).

Gut microbiota dysbiosis may contribute to cognitive impairment by increasing exposure to microbial-derived molecules and disrupting gut–brain neuroimmune communication, thereby affecting BBB integrity and neuroinflammatory pathways. However, current evidence supporting these mechanisms remains largely restricted to preclinical studies (48, 53).

#### 2.3.1 Regulation of intestinal barrier function by RhoA/ROCK signaling

The RhoA/ROCK signaling pathway is a central regulator of tight junction organization and function, and its activity is closely associated with the maintenance of epithelial and endothelial barrier integrity. Tight junctions are widely present in intestinal epithelial cells and BBB endothelial cells and consist of multiple structural components, including junctional adhesion molecules (JAMs), occludin, and claudins. Beyond serving as selective physical barriers, tight junction complexes also function as dynamic signaling platforms involved in cytoskeletal regulation, cell proliferation, differentiation, and transcriptional control (48). The activity of many transmembrane tight junction proteins relies on interactions with intracellular scaffolding molecules. For example, zonula occludens-1 (ZO-1) connects tight junction proteins with the actin cytoskeleton, thereby maintaining junctional organization and mechanical stability (56).

Excessive activation of RhoA/ROCK signaling induces actomyosin contraction and generates abnormal mechanical forces, ultimately disrupting epithelial tight junctions and adherens junctions. As a consequence, intestinal barrier integrity is compromised, leading to increased paracellular permeability, a phenomenon commonly referred to as “intestinal leakage” (57). Several molecular mechanisms contribute to this process. First, ROCK inhibits myosin light chain phosphatase (MLCP), thereby reducing myosin light chain (MLC) dephosphorylation and promoting MLC phosphorylation, which enhances actin–myosin contractility. Second, activated ROCK phosphorylates and activates LIM kinase (LIMK), resulting in suppression of cofilin activity. Since cofilin normally facilitates actin filament severing and depolymerization, its inhibition promotes F-actin stabilization and reinforces cytoskeletal contraction (58). In addition, prolonged activation of the RhoA/ROCK pathway may reduce tight junction protein expression, further weakening intestinal barrier function (59).

Conversely, insufficient RhoA/ROCK activity can also compromise barrier maintenance. Excessive inhibition or loss of RhoA/ROCK signaling disrupts cytoskeletal organization and decreases stress fiber formation, preventing the establishment of appropriate junctional tension and thereby increasing intestinal permeability (60). Certain pathogenic microorganisms, including *Helicobacter pylori* (61) and Citrobacter species (34), impair epithelial barrier integrity by releasing toxins or effector proteins that inhibit RhoA activity. This interference disrupts cytoskeletal organization and destabilizes tight junction structures (Figure 1).

#### 2.3.2 Regulation of the blood–brain barrier by RhoA/ROCK signaling

The blood–brain barrier (BBB) represents a specialized immunological and metabolic interface between peripheral circulation and the central nervous system (CNS). It is composed of non-fenestrated endothelial cells, pericytes, astrocytic endfeet, and the basement membrane (62, 63). Through selective control of molecular transport, the BBB maintains cerebral homeostasis and prevents excessive entry of circulating components and potentially harmful substances into the brain parenchyma. Increasing evidence indicates that BBB dysfunction represents an important pathological mechanism contributing to cognitive impairment. Loss of BBB integrity allows blood-derived factors, including fibrinogen and thrombin, to enter the brain, activate microglia and astrocytes, and initiate neuroinflammatory responses. Moreover, BBB disruption may interfere with the clearance of neurotoxic proteins such as amyloid-*β* (Aβ), thereby promoting abnormal protein accumulation and accelerating neurodegenerative processes. Thus, BBB impairment may contribute to cognitive decline through interacting mechanisms involving inflammation, metabolic disturbances, and neurovascular dysfunction (64).

The maintenance of BBB integrity requires coordinated regulation of multiple signaling pathways involved in cytoskeletal remodeling, junctional stability, and inflammatory control. Among these pathways, RhoA/ROCK signaling has emerged as an important regulator of actin dynamics and barrier permeability and may contribute to BBB alterations associated with dysfunction of the microbiota–gut–brain axis.

Tight junctions form the structural basis of BBB integrity by surrounding brain microvascular endothelial cells and limiting uncontrolled diffusion of hydrophilic molecules. In these endothelial cells, excessive activation of RhoA/ROCK signaling increases junctional contractility and alters the distribution of essential tight junction proteins, including Claudin-5, Occludin, and ZO-1, ultimately resulting in elevated BBB permeability. Activated RhoA stimulates downstream ROCK activity, promoting actin stress fiber formation and cytoskeletal remodeling, which disrupts tight junction structures (47), enhances endothelial contraction, and further increases barrier permeability (65). Conversely, inhibition of RhoA/ROCK2 signaling can restore actin cytoskeletal organization, prevent reductions in Claudin-5, Occludin, and ZO-1 expression, and decrease endothelin-1 and inflammatory cytokine production, thereby alleviating LPS-induced BBB impairment (66). In contrast, excessive RhoA activation promotes stress fiber formation, reduces ZO-1 localization at endothelial membranes, and aggravates BBB dysfunction (67).

BBB disruption can further intensify inflammatory responses (66), promote oxidative stress (68), and contribute to cerebral edema and neurotoxicity. However, recent findings suggest that RhoA signaling has a more complex role in BBB regulation than previously recognized. A macrophage/microglia–endothelial interaction mechanism has been identified in which ischemia/reperfusion (I/R)-induced RhoA upregulation in macrophages/microglia suppresses endothelial NLRP3-mediated pyroptosis and apoptosis, thereby reducing pro-inflammatory polarization and preserving BBB integrity (69). These observations highlight the context-dependent nature of RhoA signaling and indicate that RhoA activation is not inherently detrimental but may exert protective effects under specific cellular conditions.

Several pharmacological studies further demonstrate that BBB function can be modulated through regulation of the RhoA/ROCK pathway. For example, in a rat model of cerebral ischemia/reperfusion injury, anfibatide maintained BBB integrity by inhibiting TLR4/RhoA/ROCK signaling, reducing MLC phosphorylation, and restoring tight junction protein expression (70). Similarly, Annexin A1 alleviated Aβ42-induced BBB disruption in Alzheimer’s disease models through suppression of RhoA–ROCK signaling (71). Conversely, environmental factors may impair BBB function by activating this pathway. Diesel exhaust particles have been shown to induce inflammatory cytokine release, oxidative stress, reduction of tight junction proteins, and activation of RhoA/ROCK signaling, ultimately increasing BBB permeability (47).

Nevertheless, findings obtained from unrelated tissues or disease models should not be interpreted as direct evidence that microbiota alterations induce cognitive impairment through RhoA/ROCK activation. Instead, these studies emphasize the broad biological significance of RhoA/ROCK signaling in regulating diverse barrier systems and provide potential insights into its involvement in microbiota–gut–brain axis-associated pathological processes.

## 3 Dual regulatory roles of RhoA/ROCK signaling in barrier function

The influence of RhoA/ROCK signaling on barrier integrity is strongly shaped by cellular and physiological contexts, allowing this pathway to exert either disruptive or protective effects. Under pathological conditions, sustained activation of RhoA/ROCK generally promotes myosin light chain (MLC) phosphorylation, facilitates stress fiber formation, and enhances actomyosin contractility. These changes increase mechanical tension within cells, destabilize tight junction complexes, and ultimately lead to elevated barrier permeability. Consistently, inhibition of excessive RhoA/ROCK activation through pharmacological or genetic approaches can restore the expression and localization of critical tight junction proteins, including occludin, claudins, and ZO-1. However, RhoA activation is not invariably detrimental. Under physiological conditions, transient and spatially confined RhoA activity, such as mechanically induced “Rho flares,” promotes ZO-1 recruitment and supports local barrier repair. Therefore, the functional consequences of RhoA/ROCK signaling cannot be defined simply as protective or pathological; rather, they are determined by multiple factors, including activation intensity, signaling duration, subcellular distribution, cellular context, and the specific functions of ROCK1 and ROCK2 isoforms.

Evidence supporting the barrier-disruptive effects of excessive RhoA/ROCK activation has been obtained from NCM460 cells, IEC-6 cells, and adult grass carp models. In NCM460 intestinal epithelial cells, activation of the RhoA/ROCK pathway increases inflammatory protein expression while reducing tight junction protein levels, thereby contributing to impaired intestinal barrier function (72). Similarly, inhibition of ROCK/RhoA signaling in rat IEC-6 epithelial cells improves barrier integrity by decreasing paracellular permeability and limiting bacterial translocation (73). Consistent with these observations, studies in adult grass carp have shown that suppression of RhoA/ROCK activity enhances the expression of tight junction and adherens junction proteins (74), improves intestinal antioxidant capacity (75), reduces mucosal permeability, and helps preserve intestinal structural integrity (74, 76).

Despite these findings, RhoA/ROCK signaling can also contribute to barrier preservation by facilitating junctional adaptation and maintaining tissue homeostasis. In Xenopus epithelial cells, mechanical disruption of tight junctions induces calcium-dependent transient RhoA activation, which promotes ZO-1 recruitment and maintains paracellular barrier function (77). When focal junctional damage occurs, calcium influx through mechanosensitive channels initiates a short-lived RhoA activation event known as a “Rho flare.” This localized response promotes epithelial repair and contributes to the restoration of tissue integrity (78). In addition, adherens junctions provide essential structural support for tight junction complexes. RhoA regulates adherens junction formation through modulation of E-cadherin stability (79), whereas ROCK contributes to junctional maintenance through phosphorylation of adherens junction-associated proteins (80).

At the molecular level, the bidirectional effects of RhoA/ROCK signaling on barrier regulation are largely determined by the characteristics of upstream stimuli, activation patterns, cellular environment, and the balance between RhoGEFs and RhoGAPs. Physiological RhoA activation is typically characterized by low-intensity and transient signaling, which is necessary for maintaining cell polarity, basal junctional tension, and localized repair processes. By contrast, pathological activation is usually associated with strong, persistent, and widespread signaling induced by inflammatory stimulation, oxidative stress, or tissue injury. Thus, the distinction between beneficial and harmful RhoA activity does not rely solely on whether the pathway is activated, but rather on the magnitude, duration, spatial organization, and cellular context of the response (81).

For example, localized tight junction disruption or mechanical stretching can activate mechanosensitive Ca^2+^ channels, resulting in calcium influx and recruitment of p115RhoGEF to damaged junctional sites. This process induces a transient and spatially restricted RhoA “flare,” which promotes myosin II-mediated contraction and ZO-1 redistribution, thereby enabling rapid restoration of barrier function. In contrast, pathological stimuli, including LPS–TLR4 activation, TNF-*α* or IL-1β receptor signaling, thrombin-induced GPCR activation, and chronic oxidative stress, stimulate RhoGEFs such as GEF-H1 and drive prolonged, widespread RhoA/ROCK activation. Persistent pathway activation inhibits MLCP, increases MLC phosphorylation, promotes stress fiber formation, alters tight junction organization, and ultimately enhances barrier permeability.

The biological outcome of RhoA signaling is also closely linked to its temporal and spatial properties. Moderate activation confined to junctional regions and lasting only seconds to minutes generally facilitates junctional repair and barrier adaptation. In contrast, intense and broadly distributed activation maintained over prolonged periods is more likely to induce excessive contraction and amplify inflammatory responses. Conversely, inadequate RhoA activity may also be detrimental by impairing F-actin organization and disrupting proper tight junction assembly (82).

Furthermore, the effects of RhoA/ROCK activation differ substantially among cell types. In intestinal epithelial cells and brain microvascular endothelial cells, basal RhoA/ROCK activity contributes to cell polarity, junctional tension, and barrier stability, whereas chronic overactivation promotes cytoskeletal contraction, redistribution of junction-associated proteins, and increased permeability. In microglia and macrophages, RhoA/ROCK signaling regulates migration, phagocytic activity, and responses to injury; however, persistent activation may promote pro-inflammatory polarization and excessive production of inflammatory mediators (83). In neurons, transient RhoA/ROCK activity contributes to dendritic spine maintenance and synaptic remodeling, whereas prolonged activation is more commonly associated with growth cone collapse, axonal retraction, and synaptic dysfunction. Therefore, the role of RhoA/ROCK signaling should be interpreted within specific cellular and pathological contexts rather than being broadly categorized as either protective or detrimental (84).

Finally, the dynamic regulation of RhoA activity depends on the coordinated actions of RhoGEFs and RhoGAPs, which determine signal initiation, maintenance, and termination. Transient recruitment of junction-associated p115RhoGEF supports adaptive barrier remodeling and repair. In contrast, excessive activation of GEF-H1 or insufficient RhoGAP-mediated inactivation may shift RhoA signaling from a localized reparative response toward sustained pathological contraction and barrier dysfunction.

## 4 Regulation of neuroinflammation by RhoA/ROCK signaling

**Figure 2:**
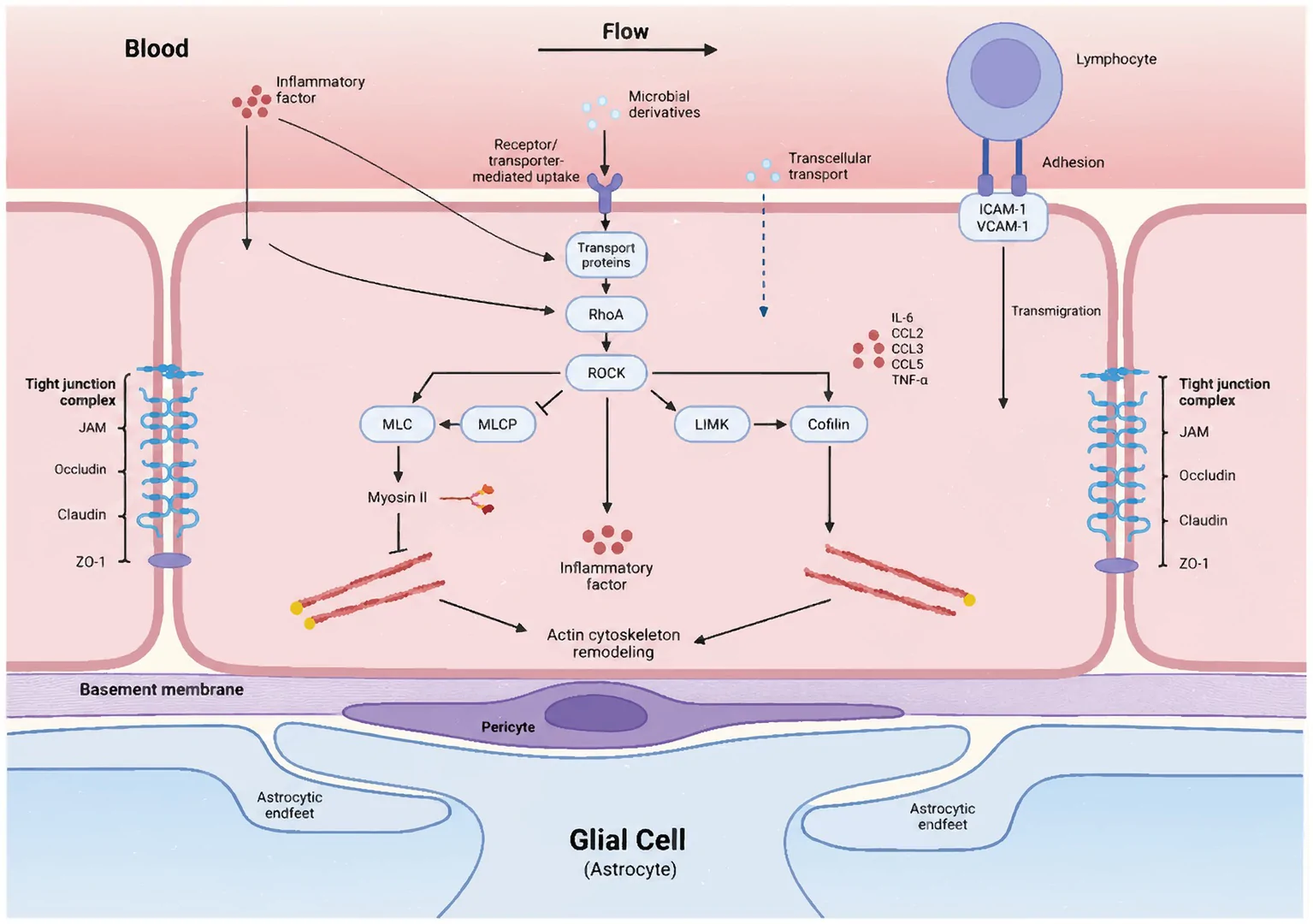
RhoAROCK signaling in microbiota-related blood–brain barrier dysfunction.

Neuroinflammation represents a complex inflammatory response within the central nervous system (CNS), primarily involving immune-related cell populations such as microglia and astrocytes. This process is widely recognized as a critical factor contributing to the onset and progression of cognitive impairment and neurodegenerative diseases (85). RhoA/ROCK signaling is involved in multiple aspects of neuroinflammatory regulation, including maintenance of blood–brain barrier (BBB) integrity, immune cell recruitment, microglial activation, and neuronal injury. Through its coordinated effects on vascular barrier function and CNS immune responses, this pathway may serve as an important regulator of neuroinflammatory processes associated with cognitive decline. The potential mechanisms by which RhoA/ROCK signaling influences BBB disruption, glial responses, and neuronal damage are illustrated in Figure 2.

Under neuroinflammatory conditions, various pro-inflammatory cytokines, including IL-1*α*, IL-1β, IL-17, IL-22, and TNF-α, can activate RhoA/ROCK signaling in endothelial cells (86). Once activated, this pathway reduces the expression of tight junction proteins, promotes endothelial cell contraction, and induces cytoskeletal remodeling accompanied by stress fiber formation, thereby increasing BBB permeability (87). Loss of BBB integrity allows circulating inflammatory factors and immune cells to enter the CNS, further amplifying inflammatory responses and creating a positive feedback loop. In addition, interactions between peripheral immune cells and endothelial adhesion molecules, such as VCAM-1 and ICAM-1, may further stimulate RhoA/ROCK activation, facilitating endothelial transmigration and enhancing inflammatory cell infiltration into the CNS (88).

Studies using microglial models have demonstrated that suppression of RhoA/ROCK signaling can reduce pro-inflammatory polarization and attenuate NF-κB-mediated inflammatory responses (89). Under normal physiological conditions, microglia function as the primary resident immune cells of the CNS and contribute to neural homeostasis. However, excessive activation of RhoA/ROCK signaling promotes microglial migration and polarization toward a pro-inflammatory M1-like phenotype (89). This transition is accompanied by enhanced production of inflammatory cytokines and neurotoxic mediators, which further aggravate neuronal injury. Increasing attention has therefore been directed toward microglial dysfunction as a key contributor to inflammation-related cognitive decline. Although microglial activation may initially serve protective functions during mild stress or early pathological challenges, prolonged activation during disease progression can result in persistent inflammation, disruption of neural networks, and deterioration of cognitive function.

Maintaining an appropriate level of RhoA activity is essential for preserving microglial survival and functional balance during inflammatory responses. Moderate RhoA activation can restrict excessive inflammatory polarization and protect against cell death, whereas loss of RhoA activity disrupts intracellular Ca^2+^ and pH homeostasis, ultimately inducing microglial apoptosis (90). Furthermore, phagocytosis of apoptotic neurons activates a TREM2–APOE-dependent transcriptional program in microglia, promoting the transition from a homeostatic phenotype toward a disease-associated microglial state. However, this phenotypic shift is highly dependent on the surrounding biological environment and should not be regarded as an inherently pro-inflammatory process (91).

In astrocytes, activation of the RhoA/ROCK pathway facilitates the acquisition of reactive phenotypes and enhances neuroinflammatory responses through increased release of inflammatory mediators. Notably, inhibition of ROCK1 has been shown to reduce tau phosphorylation and exert anti-inflammatory effects in Alzheimer’s disease models (92). Beyond its role in glial regulation, RhoA/ROCK signaling directly affects neuronal structural plasticity. Elevated RhoA activity can promote axonal and dendritic retraction, synaptic loss, and neuronal death, thereby further reinforcing inflammatory damage within the CNS (93). As a key regulator of cellular responses to injury, inhibition of RhoA/ROCK signaling has demonstrated potential in alleviating neuroinflammation and cognitive deficits following brain injury (94).

BBB disruption and neuroinflammatory activation are closely interrelated processes. Impaired BBB integrity permits blood-derived proteins, including fibrinogen, to infiltrate the brain parenchyma. Once deposited in the CNS, fibrinogen interacts with CD11b on microglia, activates the PI3K/Akt/RhoA signaling pathway, and promotes neuroinflammation and cognitive impairment in models of age-associated postpartum disease (95).

Nevertheless, RhoA/ROCK signaling should not be viewed solely as a driver of inflammatory pathology. Under physiological conditions, transient and localized RhoA activation may contribute to tissue maintenance and repair. For instance, in Xenopus epithelial cells, mechanically induced Ca^2+^ signaling activates RhoA through p115RhoGEF, enhances ZO-1 recruitment, and facilitates cytoskeletal rearrangement, ultimately restoring paracellular barrier integrity (77). This mechanism raises the possibility that physiological RhoA activity may indirectly limit neuroinflammation by maintaining intestinal barrier function and reducing systemic exposure to microbial products and inflammatory mediators. However, the protective roles of physiological RhoA activation remain incompletely understood and require further investigation.

Collectively, RhoA/ROCK signaling regulates neuroinflammatory processes through its integrated effects on BBB stability, immune cell activation, and neuronal structural plasticity. Importantly, the biological consequences of this pathway cannot be defined exclusively as pro-inflammatory or protective; instead, they are determined by cellular identity, activation dynamics, and the specific pathological context.

## 5 Regulation of neuronal and synaptic plasticity by RhoA/ROCK signaling

RhoA can be activated by various axon growth-inhibitory cues, including chondroitin sulfate proteoglycans (CSPGs), myelin-associated glycoprotein (MAG), oligodendrocyte myelin glycoprotein (OMgp), Nogo, and their corresponding receptors. Following activation, RhoA stimulates its major downstream effector ROCK, which regulates a series of cytoskeletal and signaling molecules, including LIM kinase, myosin light chain phosphatase (MLCP), collapsin response mediator protein-2 (CRMP-2), and phosphatase and tensin homolog (PTEN). Through these downstream mechanisms, RhoA/ROCK signaling collectively restricts neurite extension and limits axonal regeneration.

At the molecular level, ROCK-mediated activation of LIM kinase results in cofilin inactivation and stabilization of actin filaments within growth cones, thereby suppressing axonal elongation. Meanwhile, ROCK inhibits MLCP activity, leading to increased myosin II-dependent contractility and promoting the formation of antiparallel actin bundles within growth cones, which subsequently restricts microtubule advancement. In addition, CRMP-2 phosphorylation inhibits tubulin polymerization and further limits microtubule extension. Although RhoA/ROCK-dependent regulation of PTEN has been described in several non-neuronal cell types, direct evidence confirming this mechanism in neurons or the central nervous system (CNS) remains limited. PTEN activation may suppress Akt signaling, reduce microtubule stability and protein synthesis, and ultimately further compromise axonal regeneration (96).

As illustrated in Figure 1, RhoA activation generally acts as a negative regulator of neuronal development, differentiation, migration, and regeneration by inducing growth cone collapse and neurite retraction, thereby reducing neuronal regenerative potential (97). For example, in a lamprey spinal cord injury model, suppression of RhoA expression enhanced axonal regrowth at lesion sites and protected large reticulospinal neurons from apoptosis (98). Accordingly, pharmacological or genetic modulation of RhoA/ROCK signaling has emerged as a potential approach for promoting neural repair in various CNS disorders, including vascular dementia (99), Alzheimer’s disease (100), spinal cord injury (101), and ischemic stroke (102).

Beyond axonal regeneration, RhoA/ROCK signaling also plays a critical role in synaptic development and plasticity (103). During synapse formation, the RhoA-specific guanine nucleotide exchange factor (GEF) Ephexin5 interacts with EphB2 and activates ROCK, thereby enhancing actomyosin contractility and restricting synaptic formation and remodeling (104). Dendritic spines, as major postsynaptic structures responsible for receiving synaptic inputs, provide an essential structural basis for cognitive function. Excessive activation of RhoA/ROCK signaling increases actomyosin contraction, resulting in dendritic spine shrinkage or elimination and subsequent impairment of cognitive processes. However, during the early phase of long-term potentiation (LTP), moderate RhoA/ROCK activity may contribute to dendritic spine stabilization by inhibiting cofilin-mediated actin depolymerization, whereas excessive activation may disrupt LTP, although the underlying mechanisms remain to be fully clarified.

During long-term depression (LTD), RhoA/ROCK-dependent contractile activity facilitates internalization of postsynaptic AMPA receptors (105). The balance between contraction-promoting RhoA signaling and extension-promoting Rac1 activity is essential for maintaining appropriate synaptic remodeling during LTD. Increasing evidence supports the involvement of RhoA/ROCK signaling in synaptic plasticity regulation. In Neuro-2A cells and ICR mouse models, inhibition of the RhoA/ROCK/MLC or RhoA/ROCK/cofilin pathways alleviated pathological alterations, improved cognitive performance, and restored synaptic protein expression (106). Conversely, activation of PDZ-RhoGEF selectively decreased the density of learning-associated thin dendritic spines in mice without affecting the stability of stubby or mushroom-shaped spines (107). Moreover, inhibition of RhoA/ROCK1 signaling in hippocampal neurons reversed stress-induced reductions in dendritic spine density and abnormalities in synaptic ultrastructure, highlighting the importance of this pathway in hippocampal synaptic plasticity (108).

Genetic alterations of RhoA in mice lead to impaired synaptic transmission, reduced plasticity, and disruption of excitatory/inhibitory balance, resulting in behavioral abnormalities and altered neuronal development (45). In addition, various extracellular signals regulate synaptic plasticity through modulation of RhoA activity, including neurotransmitters such as glutamate and dopamine, pro-inflammatory cytokines, and lipid-associated receptors. For example, S1PR3 may enhance synaptic plasticity and alleviate depressive-like behaviors by suppressing RhoA/ROCK1 signaling, although the upstream regulatory mechanisms remain unclear. Interestingly, RhoA may exert distinct effects in astrocytes, where modulation of YAP signaling can reduce reactive astrocyte proliferation, thereby removing a potential barrier to regeneration and facilitating axonal growth.

ROCK consists of two isoforms, ROCK1 and ROCK2, which exhibit overlapping yet distinct biological functions. ROCK2 is relatively enriched in brain tissue and neurons, whereas ROCK1 is more broadly distributed throughout the CNS and may play a particularly important role in specific glial populations (109). Although both isoforms regulate neuronal cytoskeletal dynamics and synaptic plasticity, their functional contributions are not identical. ROCK1 preferentially regulates myosin light chain phosphorylation, stabilizes actomyosin structures, and contributes to dendritic spine neck formation and cellular polarity. In contrast, ROCK2 influences dendritic spine morphology, postsynaptic density organization, axonal stability, and injury-induced regeneration through LIMK/cofilin, Rac1, and myosin light chain-related pathways (110). Genetic studies suggest that disruption of either isoform may result in reduced dendritic spine density, impaired excitatory synaptic transmission, decreased LTP, and learning deficits, indicating that both ROCK1 and ROCK2 are required for normal synaptic function (111). However, whether gut microbiota-derived metabolites selectively influence ROCK1 or ROCK2 remains unknown. Future investigations should therefore evaluate ROCK1 and ROCK2 simultaneously, including their expression levels, subcellular localization, and kinase activity.

In addition to circulating and immune-mediated pathways, the vagus nerve represents a potential rapid communication route through which gut microbial signals reach the brain. Germ-free conditions reduce vagal afferent activity, whereas microbial colonization restores vagal function. Microbial metabolites, including short-chain fatty acids, bile acids, and indole derivatives, may activate vagal afferents and nucleus tractus solitarius neurons through FFAR2-, TGR5-, or TRPA1-dependent mechanisms (112). Administration of *Lactobacillus rhamnosus* JB-1 alters GABA receptor expression in multiple brain regions and modifies behavioral responses, while these effects are abolished after vagotomy (113, 114). Furthermore, gastrointestinal vagal afferents regulate hippocampal BDNF expression, neurogenesis, and memory formation through the “nucleus tractus solitarius–medial septum–hippocampus” pathway (115).

Despite these findings, direct evidence demonstrating that specific microbial species or metabolites regulate central RhoA/ROCK activity through vagal pathways and subsequently influence synaptic plasticity or cognitive outcomes is still lacking. Future studies should integrate region- and cell-specific RhoA biosensors, ROCK1/ROCK2 activity measurements, and rescue experiments to determine whether vagal signaling modulates central RhoA/ROCK activity through neurotransmitter- or inflammation-related mechanisms (39).

Compared with the extensive evidence supporting RhoA/ROCK regulation of neuronal cytoskeletal remodeling, direct connections among gut microbiota-derived signals, central RhoA/ROCK activity, and specific synaptic alterations remain insufficiently characterized. Current studies suggest that microbiota composition and microbial metabolites may influence RhoA/ROCK-related signaling in the brain; however, most investigations have not simultaneously examined changes in synaptic morphology, synaptic protein expression, and electrophysiological plasticity.

In chronic stress mouse models, valerate supplementation increased hippocampal GPR41 expression and suppressed RhoA/ROCK1-associated inflammatory signaling, accompanied by reductions in neuroinflammation, neuronal ferroptosis, and depressive-like behaviors. These findings provide preliminary support for a potential “microbial metabolite–GPR41–RhoA/ROCK1–brain pathology” signaling axis. Nevertheless, the causal requirement of this pathway has not been demonstrated through GPR41 inhibition, cell-specific RhoA/ROCK1 manipulation, or rescue experiments. Moreover, the behavioral outcomes primarily reflected depressive-like behaviors rather than cognitive function or synaptic plasticity.

Studies using germ-free animals and microbiota colonization models provide another possible mechanistic perspective. Microbiota depletion has been associated with altered expression of Sema3A, NRP1, NR1D1, and ROCK2 in the prefrontal cortex. Experiments using primary cortical neurons further suggest that Sema3A may regulate NR1D1 expression through RhoA/ROCK-dependent mechanisms. These findings suggest that microbiota status may influence axon guidance-related pathways and RhoA/ROCK-associated molecular alterations in the brain. However, the responsible microbial taxa or metabolites have not been identified, and microbiota restoration does not fully reverse all molecular and behavioral abnormalities. Furthermore, alterations in NR1D1 and axon guidance-related gene expression cannot be directly interpreted as mature synaptic plasticity deficits. The study also did not establish that RhoA/ROCK directly mediates changes in specific synaptic proteins, dendritic spine structures, or LTP (45).

Overall, RhoA/ROCK signaling represents a central regulatory pathway involved in axonal growth, dendritic spine remodeling, and synaptic function. However, whether it serves as a direct molecular bridge connecting gut microbiota alterations with changes in synaptic plasticity and cognitive performance remains unresolved, largely due to the current lack of comprehensive causal evidence.

## 6 Disease-specific roles of RhoA/ROCK signaling in cognitive impairment-related disorders

Cognitive impairment is a prevalent feature of several neurological disorders, including Alzheimer’s disease (AD), vascular dementia, and Parkinson’s disease (PD). Although these disorders arise from distinct pathological processes, they ultimately share a progressive decline in cognitive function. In AD, memory deterioration represents the core clinical manifestation (17), whereas cognitive deficits in vascular dementia are primarily attributed to cerebrovascular injury and subsequent neuronal dysfunction (18). In PD, dementia typically develops during the advanced stages of disease and is characterized by impairments in attention, executive function, and visuospatial abilities (19). Increasing evidence indicates that RhoA/ROCK signaling contributes to cognitive impairment across these disorders; however, its specific biological effects are largely determined by the underlying disease mechanisms, involved cell types, and pathological environment.

In AD, the major pathological hallmarks include *β*-amyloid (Aβ) deposition resulting in senile plaque formation and abnormal tau hyperphosphorylation leading to neurofibrillary tangle accumulation (17). These pathological events subsequently trigger neuroinflammation, synaptic disruption, and neuronal loss. Within this context, RhoA/ROCK signaling appears to be closely associated with both protein pathology and regulation of the synaptic cytoskeleton. Studies using human brain samples have identified elevated ROCK1 expression in individuals with AD-related mild cognitive impairment as well as in patients with AD (116). Moreover, experimental evidence indicates that Aβ42 oligomers enhance downstream ROCK–LIMK1 signaling, whereas reducing ROCK1 expression decreases Aβ accumulation in brain tissue (117). These findings suggest that Aβ may function not only as an upstream stimulus activating RhoA/ROCK signaling but also that ROCK activity may contribute to Aβ production or deposition through reciprocal regulatory mechanisms. At the synaptic level, activation of the Aβ42–ROCK2–LIMK1 pathway promotes dendritic spine degeneration and alters neuronal excitability, whereas inhibition of LIMK1 improves the ability of dendritic spines to withstand Aβ-induced structural damage.

Vascular dementia is primarily associated with cerebrovascular pathology, including chronic cerebral ischemia and hypoxia, which contribute to white matter demyelination, neuronal degeneration, and progressive cognitive decline (18). Persistent activation of RhoA/ROCK signaling promotes vascular smooth muscle contraction, reduces vasodilatory capacity, and facilitates pathological changes such as vasospasm, oxidative stress, thrombosis, and endothelial dysfunction. These vascular abnormalities further impair cerebral blood supply and aggravate chronic cerebral hypoperfusion. In a rat model of chronic cerebral hypoperfusion, treatment with the ROCK inhibitor fasudil improved cognitive performance and reduced hypoperfusion-related brain injury (118). Similarly, inhibition of the Rho/ROCK pathway in chronic cerebral ischemia models has been associated with neuroprotective effects, decreased neuronal apoptosis, and improved cognitive outcomes (119). Thus, unlike AD, the major pathological sites of RhoA/ROCK activity in vascular dementia appear to involve cerebrovascular components, including vascular walls, brain microvascular endothelial cells, and the neurovascular unit following ischemic damage.

However, the current understanding of RhoA/ROCK involvement in vascular dementia is largely based on chronic cerebral hypoperfusion models, such as bilateral common carotid artery occlusion or stenosis models. Although these experimental systems reproduce several features of vascular cognitive impairment, they do not fully capture the complexity of human cerebrovascular conditions, including cerebral small vessel disease, multiple infarctions, and mixed dementia. Therefore, whether these findings can be generalized across different vascular dementia subtypes remains to be determined through additional experimental models and clinical investigations (120).

The defining pathological feature of PD is the misfolding and aggregation of *α*-synuclein, resulting in the formation of Lewy bodies (19). This abnormal protein accumulation affects multiple neuronal populations, particularly dopaminergic and cholinergic neurons. Cognitive impairment emerges when Lewy pathology extends beyond the basal ganglia and involves cortical regions as well as cholinergic neurons in the basal forebrain. In MPTP-induced PD models, pharmacological inhibition of ROCK enhances the survival of nigral dopaminergic neurons and reduces axonal degeneration, indicating that RhoA/ROCK signaling contributes to the deterioration of dopaminergic neurons and nigrostriatal pathways (121). Consistently, fasudil treatment has been shown to alleviate dopaminergic neuronal damage and improve motor deficits in MPTP-treated animals.

Beyond neuronal degeneration, ROCK signaling also plays an important role in microglia-mediated neurotoxicity during PD progression. Activation of the angiotensin II–AT1 receptor pathway stimulates microglial RhoA/ROCK signaling, thereby enhancing MPTP-induced microglial activation and dopaminergic neuronal loss (122). These pathological effects can be attenuated by either ROCK inhibition or genetic deletion of the AT1 receptor. Furthermore, ROCK/Cdc42 signaling regulates microglial migration toward damaged dopaminergic neurons, neuron–microglia interactions, and phagocytic clearance of degenerating neurons (123). Therefore, regulation of microglial migration and phagocytic activity represents a disease-specific aspect of RhoA/ROCK signaling in PD. Nevertheless, most studies examining RhoA/ROCK in PD have focused on motor symptoms and nigral neurodegeneration, whereas it’s direct contribution to PD-associated cognitive impairment remains poorly understood. It is possible that RhoA/ROCK influences cognitive function indirectly through modulation of nigrostriatal and cortical circuits, microglial responses, and *α*-synuclein pathology; however, this hypothesis requires further validation in α-synucleinopathy models with well-characterized cognitive phenotypes.

Collectively, neuroinflammation, BBB disruption, cytoskeletal remodeling, and synaptic dysfunction represent shared mechanisms through which RhoA/ROCK signaling may contribute to cognitive disorders. Nevertheless, the relative contribution and pathological significance of these processes vary considerably among different diseases. In AD, RhoA/ROCK may primarily function as a molecular intermediary connecting Aβ-associated signaling with synaptic actin remodeling, thereby influencing dendritic spine stability, synaptic transmission, and memory formation. In vascular dementia, RhoA/ROCK appears to operate closer to the upstream vascular pathology, regulating processes such as vasoconstriction, endothelial dysfunction, cerebral hypoperfusion, and white matter injury. In PD, its major roles are associated with dopaminergic neuronal survival, axonal degeneration, microglial regulation, and *α*-synuclein-related pathological changes.

## 7 Discussion

In this review, we examined the current understanding of RhoA/ROCK signaling in cognitive impairment within the context of the microbiota–gut–brain axis. Accumulating evidence suggests that RhoA/ROCK may act as a convergent molecular pathway that integrates microbiota-associated disturbances with multiple pathological processes, including intestinal barrier impairment, systemic inflammatory responses, neuroinflammation, BBB dysfunction, and synaptic alterations, ultimately contributing to cognitive decline. Beyond its involvement in gut–brain communication, this signaling pathway may also serve as an intermediary through which microbial-derived molecules and metabolites influence both gastrointestinal homeostasis and central nervous system function. The effects of RhoA/ROCK signaling on cognition appear to be largely mediated by its regulation of barrier integrity and neuronal structural plasticity.

Despite growing recognition of the importance of RhoA/ROCK signaling in barrier regulation, several fundamental questions remain unanswered. Although moderate activation of this pathway is necessary for maintaining barrier stability, the mechanisms by which cells precisely adjust RhoA activity according to signal strength, temporal characteristics, and subcellular localization are still not fully understood. Moreover, both the intestinal barrier and BBB represent complex multicellular systems composed of coordinated interactions among endothelial cells, pericytes, astrocytes, epithelial cells, goblet cells, and immune populations. How these diverse cellular components communicate through RhoA-dependent mechanisms and achieve integrated regulation remains an important direction for future research.

A growing number of bioactive compounds have been shown to affect intestinal barrier function through modulation of the RhoA/ROCK pathway, including the traditional Chinese medicine formulation Sishen Pill (124), arsenic exposure (125), and ethanolic extracts of Atractylodis Rhizoma (126). Suppressing excessive pathological activation of RhoA/ROCK may contribute to restoration of barrier integrity, reduction of neuroinflammatory responses, and improvement of synaptic damage. However, whether broad and sustained inhibition of this pathway can be safely applied in cognitive disorders requires further investigation (127). RhoA/ROCK signaling also participates in essential physiological processes, such as vascular tone regulation, epithelial and endothelial junction maintenance, immune cell migration, axonal organization, and synaptic remodeling. Therefore, therapeutic outcomes are likely to depend strongly on intervention intensity, treatment duration, and disease context. While inhibition of abnormal RhoA/ROCK activation may provide therapeutic benefits, excessive suppression may interfere with physiological barrier repair, cerebral circulation, and synaptic processes underlying learning and memory.

Currently, commonly used ROCK inhibitors, including fasudil and Y-27632, display limited selectivity toward individual ROCK isoforms and cannot effectively distinguish between ROCK1 and ROCK2. In addition, these compounds may influence other kinase pathways when administered at relatively high concentrations (128). Systemic ROCK inhibition may also induce vasodilation and hypotension, raising particular concerns for elderly individuals and patients with vascular cognitive impairment (129). Existing evidence is primarily derived from experimental models and short-term clinical safety evaluations, whereas long-term randomized controlled trials examining cognitive outcomes are still lacking (130), as shown in Tables 1, 2. Therefore, RhoA/ROCK should be viewed as a therapeutic target requiring precise and context-dependent regulation rather than a pathway suitable for generalized and continuous inhibition. Future therapeutic strategies should emphasize the development of ROCK isoform-selective inhibitors, targeted delivery approaches based on specific brain regions or cell populations, and intermittent low-dose treatment regimens. Comprehensive assessment of cardiovascular parameters, cerebral perfusion, hepatic and renal function, pathway engagement, and cognitive performance will be essential for defining a therapeutic window that suppresses pathological signaling while preserving physiological functions.

**Table 1:** Gut microbiota and microbial-derived factors modulate the RhoA/ROCK signaling pathway: Sites of action, key effects, and cognitive outcomes.

Microbiota factor/Microbial component	RhoA/ROCK modulation	Site of action	Key effects	Cognitive outcomes	Evidence type
Short-chain fatty acids (SCFAs)	Inhibits excessive RhoA activation; downregulates ROCK1	Intestinal barrier, brain	Maintains barrier stability; suppresses neuroinflammation	Ameliorates or preserves cognitive function	In vivo / in vitro studies (7)
*Escherichia coli* (pathogenic strains)	Inactivates RhoA	Intestinal epithelium	Cytoskeletal disruption; tight junction disassembly; barrier destruction	Indirectly contributes to cognitive impairment	In vivo studies (32, 33)
Bifidobacteria-derived proteins (GroEL, transaldolase)	Modulates RhoA/ROCK signaling	Intestinal epithelium	Inhibits inflammatory cytokine production; upregulates tight junction protein expression	Indirectly supports cognitive function	Cell / in vivo studies (34)
Probiotic mixture (Limosilactobacillus fermentum HF07 + *Lactococcus lactis* HF08)	Downregulates key RhoA/ROCK pathway proteins	Intestinal tract	Alleviates inflammation; protects intestinal barrier	Indirectly supports cognitive function	In vivo studies (35)
Taurodeoxycholic acid (microbial metabolite)	Activates RhoA signaling pathway	Innate lymphoid cells	Enhances intestinal tissue residency of innate lymphoid cells; protects intestinal barrier integrity	Indirectly supports cognitive function	In vivo / cell studies (36)
LPS	Activates RhoA/ROCK via TLR4–PKC/GEF-H1 signaling	BBB endothelial cells	Tight junction disruption; increased BBB permeability; inflammatory amplification	Associated with cognitive dysfunction through BBB injury	Mechanistic + preclinical evidence (41)
Indole metabolites (tryptophan-derived)	No confirmed direct regulation of RhoA/ROCK	Intestinal epithelium, neural progenitor cells, immune cells	Enhances barrier integrity; regulates neuroimmune signaling; promotes neurogenesis	Improves cognition-related phenotypes in animal models	Preclinical evidence; indirect RhoA/ROCK relevance[42, 43]
*C. difficile* toxin A (TcdA) and toxin B (TcdB)	Inactivates RhoA	Intestinal tract	Disrupts cytoskeleton and tight junctions	Indirectly contributes to cognitive impairment	Integrative analysis / multi-source evidence (38)
Butyrate / Valerate (SCFAs)	Downregulates RhoA/ROCK1 activity	Hippocampus / brain tissue	Suppresses neuroinflammation and ferroptosis	Attenuates stress-induced depressive-like behaviors; protects cognition	In vivo studies (39)
*Pediococcus pentosaceus* LAB6 and Lactiplantibacillus plantarum LAB12 (probiotics)	Inhibits RhoA activation	Neuroblastoma cells	Inhibits amyloid-β (Aβ) production; reduces intracellular stress fiber formation	Alleviates Aβ burden in Alzheimer’s disease models	In vitro studies (40)
Gut microbiota (selected taxa deficiency)	Activates RhoGEFs	Prefrontal cortex	Promotes axonal guidance signaling	Contributes to cognitive and behavioral impairment	In vivo studies (45)
Mechanical stimuli	Transient RhoA activation (“Rho flare”)	Xenopus epithelial cells / tight junctions	Repairs paracellular barrier function	Indirectly supports cognitive function	In vitro studies (77)

BBB, blood–brain barrier; CNS, central nervous system; GAPs, GTPase-activating proteins; GDIs, guanine nucleotide dissociation inhibitors; GroEL, heat shock protein 60 homolog (chaperonin GroEL); LPS, lipopolysaccharide; MLC, myosin light chain; PKC, protein kinase C; ROCK, Rho-associated coiled-coil-containing protein kinase; ROCK1, Rho-associated coiled-coil-containing protein kinase 1; RhoA, Ras homolog family member A; RhoGEFs, Rho-specific guanine nucleotide exchange factors; SCFAs, short-chain fatty acids; TcdA, toxin A of Clostridioides difficile; TcdB, toxin B of Clostridioides difficile.

**Table 2:** ROCK inhibitors and their potential neurological applications.

ROCK inhibitor	ROCK isoform selectivity	Neurological applications / disease models	Main biological effects	Evidence level	Current limitations
Fasudil	Non-selective ROCK1/ROCK2 inhibitor (preferential inhibition of ROCK2 in some contexts)	Cerebral ischemia, stroke, Parkinson’s disease models and traumatic brain injury	Reduces vascular contraction, improves cerebral blood flow, suppresses neuroinflammatory responses, protects dopaminergic neurons, and improves neurological outcomes in experimental models	Preclinical studies; limited clinical studies in cerebrovascular disorders	Lack of ROCK isoform selectivity; long-term effects on cognition remain unclear; systemic vasodilation and hypotension may limit application
Y-27632	Non-selective ROCK1/ROCK2 inhibitor	Neuronal injury models and neurite regeneration	Promotes neurite extension, reduces cytoskeletal contraction, enhances neuronal survival and cellular plasticity	Mainly cellular and animal studies	Poor clinical translation; insufficient evidence in cognitive impairment; limited isoform specificity
SAR407899	ROCK-selective inhibitor (higher selectivity than classical inhibitors)	Vascular dysfunction, experimental cerebrovascular disorders	Improves endothelial function, reduces vascular remodeling, and modulates ROCK-dependent cytoskeletal responses	Preclinical studies	Neurological applications remain insufficiently investigated
GSK269962A	Potent ROCK1/ROCK2 inhibitor	Neuroinflammation and vascular-related disease models	Suppresses ROCK-mediated inflammatory signaling and cytoskeletal remodeling	Preclinical studies	Limited data regarding CNS penetration and neurological outcomes

CNS, central nervous system; ROCK, Rho-associated coiled-coil-containing protein kinase; ROCK1, Rho-associated coiled-coil-containing protein kinase 1; ROCK2, Rho-associated coiled-coil-containing protein kinase 2.

Currently available ROCK inhibitors, including fasudil and Y-27632, generally lack sufficient selectivity between ROCK1 and ROCK2. Although preclinical studies suggest potential neuroprotective effects through regulation of vascular function, neuroinflammation, and neuronal cytoskeletal remodeling, direct clinical evidence supporting their efficacy in cognitive impairment remains limited. Future development of isoform-selective ROCK inhibitors and targeted delivery strategies may improve therapeutic precision.

From a translational perspective, fasudil currently represents the primary ROCK inhibitor undergoing clinical investigation in neurodegenerative disorders. The Fasudil Trial for Early Alzheimer’s Disease (FEAD, NCT06362707) is an ongoing randomized, double-blind, placebo-controlled phase II clinical study designed to recruit up to 200 patients with early-stage AD. Participants receive oral fasudil treatment for approximately 12 months, with working memory and episodic memory selected as the primary cognitive endpoints. Additional evaluations include other cognitive domains, cerebral glucose metabolism, and disease-associated biomarkers (131).

The completed phase II randomized controlled ROCK-ALS trial demonstrated that 20 days of intravenous fasudil administration in 120 patients with amyotrophic lateral sclerosis showed safety and tolerability comparable to placebo, with no treatment-related serious adverse events or deaths. These results support the clinical feasibility of further exploring ROCK inhibition in neurological disorders; however, they do not provide evidence for disease-modifying effects or cognitive improvement (132). Furthermore, the selective ROCK2 inhibitor belumosudil has completed phase II clinical evaluation and has entered clinical use for chronic graft-versus-host disease, demonstrating the practical feasibility of orally administered, isoform-selective ROCK inhibition (133). Nevertheless, its ability to penetrate the BBB and its potential efficacy in cognitive disorders remain unclear.

Accordingly, future clinical trials investigating RhoA/ROCK modulation in cognitive impairment should incorporate not only conventional cognitive assessments and neuroimaging approaches but also comprehensive biological profiling. Such evaluations should include fecal metagenomic and metabolomic analyses, measurement of short-chain fatty acids and bile acids, assessment of intestinal barrier integrity and endotoxin exposure, BBB-related biomarkers, and direct indicators of RhoA/ROCK pathway activation. Comparative investigations of ROCK1-selective and ROCK2-selective inhibition strategies will also be necessary to clarify their respective therapeutic advantages.

Based on currently available evidence, several experimentally testable hypotheses can be proposed. First, microbial-derived signals are unlikely to regulate RhoA/ROCK signaling through a universal mechanism. Instead, specific microbial molecules may selectively influence ROCK1 or ROCK2 activity in intestinal epithelial cells, brain microvascular endothelial cells, glial cells, or neurons depending on receptor expression and cellular context. Such cell-specific modulation may ultimately determine the consequences for barrier function and synaptic plasticity. This hypothesis can be examined using germ-free models, defined microbiota systems, stable isotope tracing, receptor-deficient approaches, and tissue-specific manipulation of RhoA/ROCK signaling.

Second, the physiological and pathological consequences of RhoA/ROCK activation are likely determined by the intensity, duration, and spatial distribution of signaling events. Transient and localized activation at cellular junctions may facilitate barrier restoration, whereas prolonged and widespread activation may drive excessive cytoskeletal contraction, inflammatory amplification, and synaptic dysfunction. Future studies combining real-time RhoA biosensors with temporally controlled optogenetic or chemogenetic approaches may provide deeper insight into how distinct activation patterns shape barrier function and cognitive outcomes.

## 8 Conclusion

With the accelerating aging of the global population, cognitive impairment and related neurodegenerative disorders have become major public health challenges, and their increasing disease burden and socioeconomic impact highlight the urgent need to elucidate underlying mechanisms and identify novel preventive and therapeutic targets. Cognitive impairment represents a complex pathological condition involving multiple interconnected mechanisms, including neuroinflammation, blood–brain barrier (BBB) disruption, synaptic dysfunction, and abnormal accumulation of disease-associated proteins. The microbiota–gut–brain axis has emerged as an important framework for understanding how alterations in intestinal microbial ecosystems influence central nervous system function. Given its extensive regulatory roles in cytoskeletal remodeling, barrier maintenance, immune responses, and neuronal plasticity, RhoA/ROCK signaling may represent a potential integrative pathway connecting microbiota-derived signals with cognitive alterations (83).

However, direct evidence demonstrating that intestinal microbiota disturbances induce cognitive impairment through abnormal regulation of RhoA/ROCK signaling remains insufficient. Current findings are mainly derived from cellular experiments and animal models, indicating that microbial metabolites and inflammation-related factors can influence RhoA/ROCK activity, while RhoA/ROCK signaling participates in neuroinflammation, BBB dysfunction, and synaptic impairment. Nevertheless, the complete causal relationship linking microbiota dysbiosis, RhoA/ROCK dysregulation, and cognitive decline has not yet been established (134). Therefore, RhoA/ROCK should currently be considered a biologically plausible candidate pathway involved in microbiota–gut–brain axis-associated cognitive dysfunction rather than a confirmed central mediator.

Furthermore, the biological consequences of RhoA/ROCK activation are highly dependent on cell type, temporal dynamics, and pathological context (29). Localized and transient activation may support barrier restoration and neuronal stability, whereas prolonged and excessive activation may contribute to persistent inflammation and neuronal injury (81). Future research integrating cell-specific molecular interventions, ROCK isoform-selective targeting strategies, multi-organ analyses, and longitudinal human studies will be essential for clarifying the causal relationships among microbiota alterations, RhoA/ROCK regulation, and cognitive impairment (128). Although current evidence remains largely at the preclinical stage, RhoA/ROCK signaling represents a promising avenue for further investigating how the microbiota–gut–brain axis contributes to cognitive disorders.
